# Impact of Perceived Severity of COVID-19 (SARS-COV-2) on Mental Health of University Students of Pakistan: The Mediating Role of Muslim Religiosity

**DOI:** 10.3389/fpsyt.2021.560059

**Published:** 2021-08-02

**Authors:** Muhammad Saleem, Abou Bakar, Areeha Khan Durrani, Zubair Manzoor

**Affiliations:** ^1^Department of Applied Psychology, The Islamia University of Bahawalpur, Bahawalpur, Pakistan; ^2^Department of Management Sciences, The Islamia University of Bahawalpur, Bahawalpur, Pakistan

**Keywords:** COVID-19, perceived severity, mental health, muslim religiosity, Pakistani students

## Abstract

**Background:** Perceived severity of COVID-19 (SARS-COV-2) is known to be associated with mental health of people in general and health professionals in particular in Western societies. However, its association with the mental health of students in Pakistan, which is predominantly a Muslim society, remains unclear so far. Moreover, the role of Muslim religiosity for such an association has not yet been investigated. We aimed to examine the association and report findings on the impact of perceived severity on mental health with a sample of students from all five provinces of Pakistan.

**Methods:** We did a cross-sectional online survey from 1,525 Pakistani students in March 2020 using standardized measurement tools. We then determined the prevalence of perceived severity among students and its impact on their mental health. The strength of associations between these variables was estimated using generalized linear models, with appropriate distribution and link functions. Structural equation modeling through SmartPLS (3.0) software was utilized to analyze the results.

**Findings:** The perceived severity of COVID-19 is significantly associated with mental health of Pakistani students, whereas Muslim religiosity is a strong mediator between perceived severity and mental health of Pakistani students.

**Conclusions:** Though the perceived severity of COVID-19 is associated with mental health, this relationship can be better explained by the role of Muslim religiosity. When tested individually, the perceived severity accounted for only 18% variance in mental health that increased up to 57% by the mediating role of Muslim religiosity. This difference clearly indicates the mediating role of Muslim religiosity in the association between perceived severity and mental health for Pakistani students.

## Introduction

At end of December 2019, an outbreak of respiratory disease cases in Wuhan, China, is caused by virus spread through the local wet market. Initially, this disease was named as Severe Acute Respiratory Syndrome Corona Virus 2 (SARS-COV-2) that later was renamed as Corona Virus Disease 2019 (COVID-19) by WHO, on February 11, 2020 (1, 2). This epidemic is spreading rapidly all over the world including many other European and Asian countries (3). This wide spreading of COVID-19 and increase in death tolls have caused extreme effect on the mental health of the whole world (4–6). Due to this outbreak, gatherings have been prohibited all over the world with emphasis on social distancing and self-quarantine, which have resulted in various depressive states, low mood, sense of loneliness, worry, and anxiousness. Studies have pointed that perceived severity of disease causes anxiousness and depression in individuals (4). This is in line with past studies that found that an individual's negative perceptions about the incident (e.g., risk and threat/severity) were related to more mental health problems during the outbreak of the severe acute respiratory syndrome (SARS) and the Ebola (7, 8). It has been reported that pandemics such as COVID-19 have caused several mental issues such as stress and negative psychiatric illness among individuals. A study conducted in Latin America (Chile) have shown that serious mental health problems are caused by the COVID-19 pandemic (9). Another study has explored that, in South Asia, the long prevalence of this pandemic COVID-19 has created several mental health-related problems (10). However, little is known about the influence of perceived severity of COVID-19 on mental health (11). On February 26, two cases of COVID-19 were confirmed in Pakistan, and according to Daily Situation Report-Pakistan-COVID-19 of May 7, a total of 24,644 cases were reported as positive for COVID-19, from which 6,464 were discharged after recovery, 181 are in critical condition, and 585 have died (Humanitarian Response Pakistan, 2020). It is an alarming state for Pakistan, being an underdeveloped country, with limited medical resources and facilities. With such rapid increase in the rate of suspected cases of COVID-19, there is great expectancy for Pakistani population to perceive the threat and fear regarding the severity of this respiratory disease as serious and significant. As discussed above, since the effect on mental health due to this pandemic is not explored to the best of authors' knowledge, this study sought to measure this effect in the Pakistani population. Furthermore, Pakistan is an Islamic country, where most of the Muslims have strong religious beliefs. They practice religion strictly and keep faith in praying and seeking help from God. In such severe situation, where the number of COVID-19-positive patients is growing each day, having strong faith on the power of the Creator could serve as a resilient factor for individuals. Having strong religious beliefs could serve as the mediator between the perceived severity for pandemic and mental health of individuals. It is a common observation that the Pakistani population tends to utilize the religious paradigm as a protective shield against any worse condition and situation as compared to other external worldviews. Previously, a number of studies have also found that religion plays a significant role in improving mental health problems such as anxiety, depression, stress, and hopelessness (12, 13). Studies have also established the fact that individuals use religion to cope with adversities. People have a firm belief that practicing several religious activities such as prayers, five times a day (Salah), helps them to relieve anxiety and depressive states and keep them hopeful for better outcomes (14, 15). Muslims often turn to their religious beliefs to cope with any loss due to incidents, and according to more recent research, religiosity is related to less depression, less anxiety, and greater well-being. Muslims who accept and hold on to teachings of religion appear to have better mental health (16). So, keeping in view the mentioned studies, this study is designed to bridge the research gap in the literature of perceived severity of COVID-19, effects on mental health, and the role of Muslim religiosity in mediating the relationship between the two. The population of this study consisted of students (young adults) as they are most vulnerable toward the perceived severity/threats of disease and mental health problems nowadays, because of limited life activities due to the lockdown situation in this COVID-19 outbreak, and because they are facing a number of stressors as compared to people of other age groups. Furthermore, the researchers had direct contact with the students, and in this state of adversity, it was crucial to address the mental health of students. So, this study was sought to examine the effect of perceived severity of COVID-19 on the mental health of Pakistani students. Moreover, this study examined the mediating role of Muslim religiosity between perceived severity of COVID-19 and mental health of Pakistani students. This study would be unique in its nature as there is no other literature to date that addresses the psychological and mental health issues regarding COVID-19 in a Pakistani perspective.

## Materials and Methods

### Design and Participants

A cross-sectional research design was employed in which data were collected from students by employing a convenient sampling technique. This sampling technique was chosen because of the emergency state of the COVID-19 pandemic where all the educational institutes were closed. The sample size was justified by utilizing the *a priori* online statistical calculator for structural equation modeling, in which anticipated effect size = 0.1, desired statistical power level = 0.9, latent variable = 2, and observed variable = 1 with 0.05 probability level. The minimum sample size calculated was 1,267 with 20% attrition rate; the sample size estimated for this study was 1,520 (17). We did an online survey through the www.questionstar.com survey website and distributed the survey link to students all over Pakistan through WhatsApp and Facebook groups. The students were from various universities and colleges of all provinces in Pakistan, but our focus was to gather information only about the province from which they belong. For this study, samples from five provinces (Punjab, Sindh, Balochistan, Khyber Pakhtunkhwa, and Gilgit-Baltistan) of Pakistan were taken. The survey questionnaire consisted of information related to research purpose, demographic information sheet, consent form and measurement instruments. A total of 1,645 students accessed the questionnaire link, among which 1,525 students completed the survey and the rest of the questionnaires were omitted because they were incomplete. The frequency distribution of the respondent's demographic characteristics is presented in Table 1 under *Results* section.

**Table 1:** Prevalence of mental health disorders (depression and anxiety) using PHQ (*N* = 1,525).

**Subscales**	**Categories**	***f* (%)**
Depression^*^	Mild	923 (60.5)
	Moderate	515 (33.8)
	Severe	87 (5.7)
Anxiety^**^	Mild	844 (55.3)
	Moderate	567 (37.2)
	Severe	114 (7.5)

* *Depression; feeling down, depressed, hopeless, and little interest in doing things*.

** *Anxiety; feeling nervous, anxious, and not able to control worrying*.

### Measurement Instruments

The online survey consisted of a set of questionnaires including consent form, demographic information sheet (age, gender, and province), and three measurement tools whose details are given below.

#### Risk Behavior Diagnostic Scale

This risk behavior diagnostic scale is used to measure the perceived severity of disease (18). It has three items that can be used to measure the perception of severity associated with any health threat. For this study, perception of COVID-19 health threat was asked from the participants, and items were as follows: (1) I believe that the health threat of COVID-19 is severe, (2) I believe that the health threat of COVID-19 is serious, and (3) I believe that the health threat of COVID-19 is significant. This scale has a five-item response scale from strongly disagree to strongly agree. The Cronbach's alpha = 0.90 for the original scale, and for the current study, the Cronbach's alpha value was 0.80.

#### Patient Health Questionnaire-4

The PHQ-4 is a four-item inventory rated on a four-point Likert-type scale (19). Its purpose is to allow a very brief measurement of depression and anxiety. According to this measurement instrument, depression is described as feeling down, depressed, hopeless, and little interest in doing things, whereas anxiety is described as feeling nervous, anxious, and not able to control worrying. The Cronbach's alpha for the current study is 0.84.

#### Muslim Religiosity Personality Inventory

The Islamic worldview subscale of Muslim Religiosity Personality Inventory (MRPI) developed by Krauss and Hamzah (20) was used in this study. This subscale has 23 items with a five-point Likert response format (strongly agree to strongly disagree). For the Islamic Worldview scale, the Cronbach's alpha was 0.86 for original scale, whereas for the internal consistency of this scale, Cronbach's alpha for the current study was 0.81.

### Procedures

A proposal for the present study was approved by the institutional research board and research process was started. It was quite feasible for the researchers to collect required data through the online survey form, so a questionnaire was developed on the web consisting of a consent form, a demographic information sheet, and questionnaires chosen for the study after receiving formal permission from the authors of measurement instruments. The respondents were informed that their participation is voluntary and the information they provided will be kept confidential. They were also ensured about the anonymity of their identities. The respondents were aware that they can leave the survey at any time they wished.

### Statistical Analysis

We analyzed data in SPSS (24.0) and SmartPLS (3.0). First, descriptive statistics for frequency distribution of demographic variables were obtained. SmartPLS was used to analyze the direct effect of latent variable (perceived severity of COVID-19) and indirect effect through a mediator (Muslim religiosity) on the dependent variable (mental health) through several steps. First, construct reliability was assessed by composite reliability analysis, and Cronbach's alpha value and average variance were estimated. Second, the construct reliability and item validity were determined using a discriminant and convergent validity method following the Fornell and Larcker (21). Third, the structural model was assessed by examining the path coefficients using standardized betas (β), sample mean, standard deviations, and *t* statistics (*t* > 1.96). Fourth, the mediating effect was used to determine the difference in the results of direct effect and indirect effect with mediating variable. In the fifth step, the value of *R*^2^ was used as an indicator of the overall predictive strength of the model. In the sixth step, the value of *f* ^2^ was used as a measure to determine the effect size of predicting variable in the model. Finally, the value of *Q*^2^ was used as a criterion to assess the model's predictive relevance (22).

## Results

This section demonstrates the tables defining the values obtained by various statistical tests and brief descriptions of study findings.

### Prevalence of Depression and Anxiety Among the Sample

Table 1 demonstrates the prevalence of mild, moderate, and severe levels of depression and anxiety among the students through frequencies and percentages.

### Sample Characteristics

In Table 2, the baseline characteristics of students are demonstrated with age, gender, and province.

**Table 2:** Baseline characteristics of study respondents (*N* = 1,525).

**Respondent's characteristics**		***f* (%)**
Age	15–18 years	541 (35.6)
	19–21 years	625 (40.9)
	21 and above	359 (23.5)
Gender	Male	749 (49.1)
	Female	776 (50.9)
Province	Punjab	451 (29.6)
	Sindh	333 (21.8)
	Balochistan	308 (20.2)
	KPK	255 (16.7)
	Gilgit-Baltistan	178 (11.7)

### Reliability and Validity Estimate of Constructs

Table 3 depicts the reliability and validity of constructs, as it is clearly seen that Cronbach's alpha of all construct lies in the acceptable range of internal consistency. The values of composite reliability and Cronbach's alpha were >0.8, indicating that the instrument used in this study showed high internal consistency (23, 24). In Table 4, the discriminant validity of the constructs has been established through cross-loadings and the Fornell–Larcker criterion method.

**Table 3:** Reliability and validity estimates of study variables.

**Variables**	**Average**	**Cronbach's alpha**	**Composite reliability**
Perceived severity of COVID-19	0.699	0.80	0.82
Mental health	0671	0.84	0.89
Muslim religiosity	0.570	0.81	0.80

**Table 4:** Discriminant validity according to the Fornell–Larcker criterion.

	**Perceived severity**	**Mental health**	**Muslim religiosity**
Perceived severity	0.670	0.421	0.96
Mental health	–	0.819	–
Muslim religiosity	–	0.703	0.755

### Perceived Severity of Disease and Mental Health

**Figure 1:**
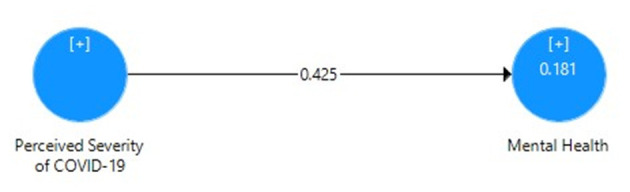
Path coefficient model for direct effect of perceived severity of COVID-19 on mental health.

**Figure 2:**
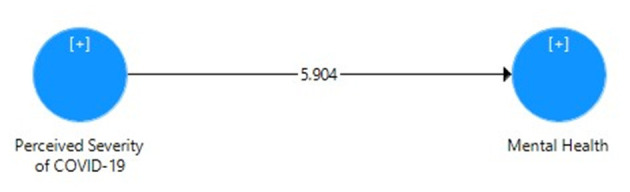
Bootstrapping model for direct effect of perceived severity of COVID-19 on mental health.

**Figure 3:**
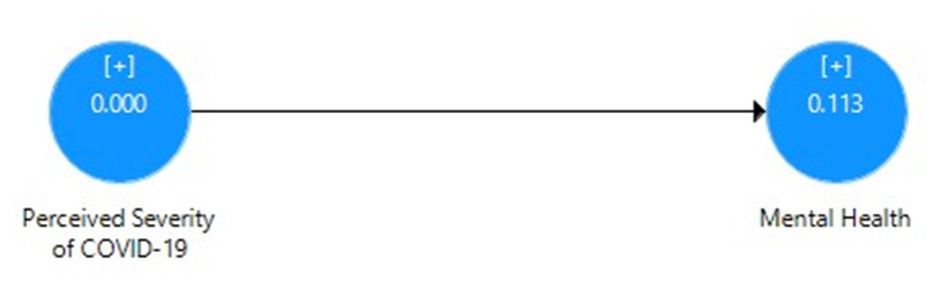
Blindfolding value for relevance of direct effect model.

Table 5 displays the direct effect of perceived severity of COVID-19 on the mental health of Pakistani students with the correlation value, mean, SD, *t*-value, *p*-value, *R*^2^, effect size, and cross-validated redundancy estimates. It can be seen that the path coefficient value β = 0.425 shows significant positive relationship and direct impact *R*^2^ = 0.18 (18% variance) of perceived severity and mental health through the PLS algorithm (Figure 1), whereas the significance of path coefficient through *t* = 5.904 at 1% level of significance was obtained through bootstrapping (Figure 2). The cross-validated redundancy and relevance of the predicted effect through *Q*^2^ = 0.113 value of model were generated from the estimates of blindfolding (Figure 3).

**Table 5:** Significance of path coefficients for perceived severity of COVID-19 > mental health.

**Direct effect of perceived severity of COVID-19 and mental health among Pakistani students (** ***N*** **=** **1,525)**									
**Relationship**	**Path coefficient**	**Mean**	**SD**	***t*** **-value**	***p*** **-value**	***R*** ^**2**^	**Adj**. ***R***^**2**^	***f*** ^**2**^	***Q*** ^**2**^
PS > MH	0.425	0.462	0.072	5.904^***^	0.000	0.181	0.172	0.221	0.113

*PS, Perceived Severity; MH, Mental Health;*

*** *Significance at 1%*.

### Muslim Religiosity as a Mediator

**Figure 4:**
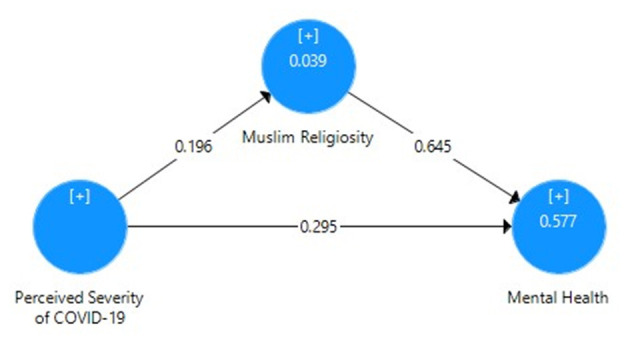
Path coefficient model for the mediating role of Muslim religiosity.

**Figure 5:**
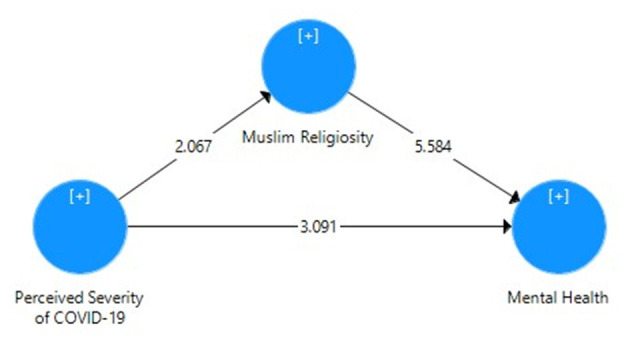
Bootstrapping model for the mediating role of Muslim religiosity.

**Figure 6:**
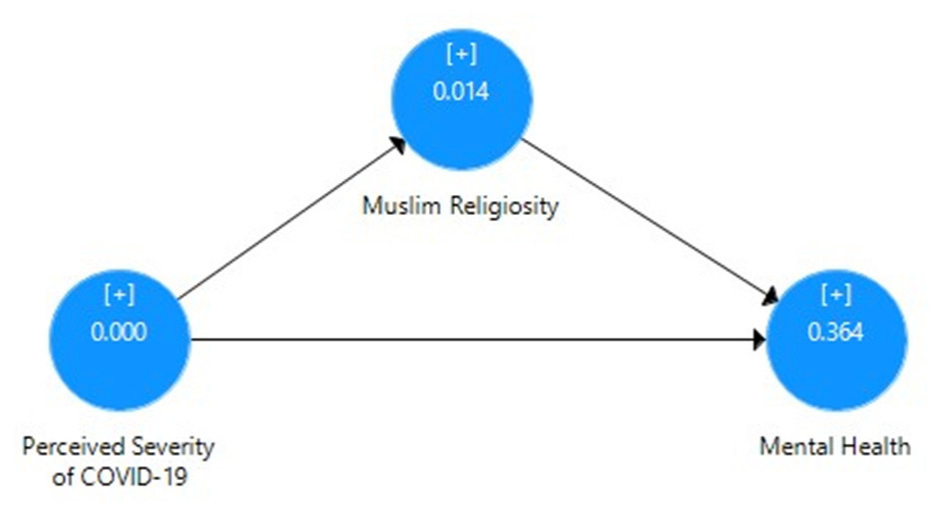
Blindfolding value for the relevance of the mediating model.

Table 6 mainly focuses on the mediating role of Muslim religiosity between perceived severity of COVID-19 and mental health of Pakistani students with the correlation value, mean, SD, *t*-value, *p*-value, *R*^2^, effect size, and cross-validated redundancy estimates. Here, it can be clearly seen that the direct relationship between perceived severity and mental health has been reduced due to the mediating role of Muslim religiosity. As shown in Figure 4, the path coefficient for perceived severity and mental health was β = 0.295, that for perceived severity and Muslim religiosity was β = 0.196, and that for Muslim religiosity and mental health was β = 0.645. These path coefficients show a significant positive relationship between these three variables. Figure 5 also depicts the indirect impact of perceived severity on mental health through Muslim religiosity with *R*^2^ = 0.577 (57% variance). Moreover, the significance of path coefficient was determined by *t*-value at 1% level of significance through bootstrapping. *Q*^2^ = 0.364 showed the cross-validated redundancy and relevance of predicted mediation, and the estimates of this *Q*^2^ (blindfolding) can be clearly seen in Figure 6.

**Table 6:** Significance of path coefficients for perceived severity of COVID-19 > Muslim religiosity > mental health.

**Indirect effect of perceived severity of COVID-19 on mental health through Muslim religiosity among Pakistani students (** ***N*** **=** **1,525)**									
**Relationship**	**Path coefficient**	**Mean**	**SD**	***t*** **-value**	***p*** **-value**	***R*** ^**2**^	**Adj**. ***R***^**2**^	***f*** ^**2**^	***Q*** ^**2**^
PS > MH	0.295	0.293	0.095	3.091^***^	0.002	-	-	-	-
PS > MR	0.196	0.231	0.095	2.067^***^	0.009	-	-	-	-
MR > MH	0.645	0.648	0.11	5.584^***^	0.000	-	-	-	-
PS > MR > MH	-	-	-	-	-	0.57	0.56	0.94	0.364

*PS, Perceived Severity; MH, Mental Health; MR, Muslim Religiosity;*

*** *Significance at 1%*.

## Discussion

COVID-19 (SARS-COV-2) is a virus that can lead toward severe respiratory disease. The vaccine for this virus is still under trial process. There are several other preventive measures suggested apart from the vaccine; for example, it can be controlled through social distance, mask-wearing, and sanitization or self-hygiene. After its rapid growth in more than 174 countries including Pakistan, every individual is concerned about its uncertainties. The severity and seriousness of COVID-19 are perceived by individuals, affecting their mental health. To evaluate this effect on Pakistani students, we used a cross-sectional research design and collected data through already developed questionnaires asking about the perceived severity of the COVID-19 pandemic, mental health, and Muslim religiosity for measuring the mediating role of religiosity in Pakistani students among predictors and predicted variables.

In the study, at first, we hypothesized that there would be a significant positive impact of perceived severity of COVID-19 on mental health of Pakistani students. The findings showed that perceived severity explained variance in mental health and there was a significant positive relationship between perceived severity and mental health of Pakistani students. Therefore, we can conclude that our hypothesis is supported, as the results show that perceived severity is a strong predictor of mental health. Extending the testing of hypothesis in this research, *f* ^2^ depicts the test for effect size and *Q*^2^ shows that the predictive relevance of the independent variable (perceived severity of COVID_19) was obtained through bootstrapping and blindfolding procedures, respectively (22). The direction of models and values within the tables helps us to understand how perceived severity of SARS-COV-2 could spread across the Pakistani population and what effects they may have on the mental health of students. The transmission of COVID-19 is itself an anxiety- and depression-inducing indicator, and it can severely affect the overall normal mental functioning. So, here we can conclude that the findings are in line with previous literature that suggested that perceived severity of COVID-19 may impose a significant main effect on mental health (25, 26). We must admit that little is known about the effect of disease severity and risk factors affecting mental health and the literature in this regard is scarce. However, the relationship established in this study between the perceived severity of disease and mental health of students can however the concerns toward other subsiding factors of health too.

Furthermore, we also hypothesized in this research that there would be a significant mediating role of Muslim religiosity between perceived severity of COVID-19 and mental health among Pakistani students. The mediation analysis shown in Table 5 shows that the perceived severity and inclusion of Muslim religiosity explains 57% of variance in mental health, indicating that the mediating variable provides moderate support for the model (27). It can be compared with the direct effect of perceived severity on mental health with 18% of variance as discussed in the outcomes of hypothesis. The hypothesis testing further shows that the relationship between perceived severity and Muslim religiosity was significantly correlated with mental health (β = 0.425, *t* = 5.904). This supports our hypothesis, and these results confirm that Muslim religiosity acts as a significant mediating variable in the relationship between perceived risk of contracting infectious disease and mental health of Pakistani students examined in this study. The findings of mediation analysis establish the notion in literature that Muslim religiosity is a significant mediator between the perceived severity of acquiring an infectious disease (i.e., COVID-19) and mental health. We can relate these findings to the previous studies, which exhibited a strong relationship between the religiosity and mental health of individuals (16, 28) and religious practices prevent individuals from mental health problems like depression, anxiety, and hopelessness (14, 29). Although much is unknown in the literature about the indirect link of disease severity on mental health through religiosity, our study has provided some support to conclude that for the Pakistani Muslim population, the significance of Muslim preaching and theological doctrines can help provide a protective shield to its followers against any state of adversity and uncertainty. We arrived at the finding that individuals who have a higher level of Muslim religiosity depicted a lower level of mental health issues and lower perceived severity of disease (COVID-19).

## Conclusion

In conclusion, our study shows that Islamic religiosity is a strong coping mechanism for Muslims against anxiety or depression The COVID-19 pandemic and its severity are affecting the mental health of individuals.

### Future Avenues of Study

Considering the emergency situation involving COVID-19 in Pakistan, we designed this study to address the possible psychological impacts in general and mental health issues in particular among the young Pakistani population. The findings of our study could help to bridge the gap regarding the psychological risk factors of COVID-19 in the existing body of knowledge. There should be further rigorous scientific investigations into aspects of religious practice that help preserve mental health in the face of adversity and the vicissitudes of life. It is also suggested to replicate the study among frontline medical workers such as doctors and allied medical staff.

### Limitations and Strengths of the Study

Being a startup research for COVID-19, there are several limitations of this study; being an initial study, the evidence for literature support was not adequate. Since COVID-19 is spreading in many countries and is considered an urgent emergency for public health, we expect to extend and improve our study taking data from other countries and improving the findings of our study. However, the results of this study can serve as preliminary evidence for future research. Furthermore, this study also holds a strength of including multi-centered data collection and a large sample size.

### Implications of the Study

Muslim religiosity plays an important part not only in improving the mental health of Pakistani students but also in the association between perceived severity of COVID-19 (SARS-COV-2) and the mental health of students. The findings contribute substantially to the understanding and management of mental health in Pakistan. In order to reduce the adverse effect of the prevailing COVID-19 pandemic on mental health, the policymakers and media in the country can appeal to the religious belief system of Pakistani people. The study also offers an initial platform for further research into exploring the social and cultural values of a society to combat such devastating situations.

## Data Availability Statement

The raw data supporting the conclusions of this article will be made available by the authors, without undue reservation.

## Ethics Statement

The studies involving human participants were reviewed and approved by Departmental Research Committee from the Department of Applied Psychology-Bahawalnagar Campus-The Islamia University of Bahawalpur. Written informed consent to participate in this study was provided by the participants' legal guardian/next of kin.

## Author Contributions

MS conceived the study and finalized the manuscript. AB did data collection and manuscript reviewing. AD did statistical analyses and manuscript writing. ZM did literature review. All authors contributed to the article and approved the submitted version.

## Conflict of Interest

The authors declare that the research was conducted in the absence of any commercial or financial relationships that could be construed as a potential conflict of interest.

## Publisher's Note

All claims expressed in this article are solely those of the authors and do not necessarily represent those of their affiliated organizations, or those of the publisher, the editors and the reviewers. Any product that may be evaluated in this article, or claim that may be made by its manufacturer, is not guaranteed or endorsed by the publisher.
