# Supplementary material for: Impact of Perceived Severity of COVID-19 (SARS-COV-2) on Mental Health of University Students of Pakistan: The Mediating Role of Muslim Religiosity
Source: Front Psychiatry. 2021 Aug 2;12:560059. doi: 10.3389/fpsyt.2021.560059 (PMC8365036; doi:10.3389/fpsyt.2021.560059)
Supplement: Supplementary file 1 [file Data_Sheet_1.PDF]

Extended Parallel Process Model

General measure. Witte and colleagues developed the Risk Behaviour Diagnosis Scale (Witte et al., 1996) which was designed to measure the components of the EPPM. The perceived severity scale has three items which can be used to measure the severity associated with any health threat. One limitation with this measure is that the items do not assess how severe the health threat would be for the individual. Another potential limitation is that the items are generic rather than disease-specific, and the lack of content specificity may reduce the predictive utility of the scale. As with all the other measures listed here, no behavioural context is specified.

1) 'I believe that [health threat] is severe'

2) 'I believe that [health threat] is serious'

3) 'I believe that [health threat] is significant'

Five item response scale: strongly disagree to strongly agree.

Cronbach's alpha= 0.90. This value was observed using a random sample of 179 women at a large university in the Midwestern United States. The majority were aged 17 to 22 (91%) and white (78.5%).
