# Supplementary material for: Impact of Perceived Severity of COVID-19 (SARS-COV-2) on Mental Health of University Students of Pakistan: The Mediating Role of Muslim Religiosity
Source: Front Psychiatry. 2021 Aug 2;12:560059. doi: 10.3389/fpsyt.2021.560059 (PMC8365036; doi:10.3389/fpsyt.2021.560059)
Supplement: Supplementary file 2 [file Data_Sheet_2.PDF]

2016

The Muslim Religiosity-Personality Inventory (MRPI) Scoring Manual

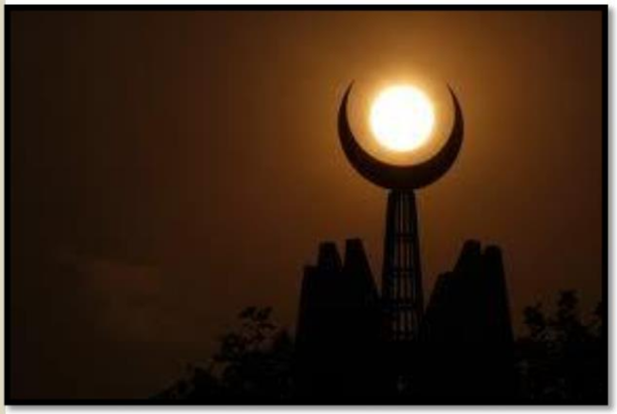

Steven Eric Krauss (Abd. Lateef)
Azimi Hamzah
Institute for Social Science Studies (IPSAS)

UPM
UNIVERSITI PUTRA MALAYSIA

APPENDIX A: The Islamic Worldview Scale

Based on the statements below, please identify the view that most accurately reflects your own by marking (**√**) the correct box

	Item	Strongly Agree	Somewhat Agree	Undecided	Somewhat Disagree	Strongly Disagree
1.	A man should leave his job when told by the doctor that he will die within a short time.					
2.	Allah S.W.T. will not forgive people who commit sins intentionally.					
3.	Damage and destruction that occur in the world are the negative results of non-believers' actions.					
4.	People are far from Allah S.W.T. when they commit sins.					
5.	All laws/rulings in the Qur'an are for the advantage and well-being of Muslims only.					
6.	Worldly life cannot be separated from life hereafter.					
7.	All deeds performed by people who have reached the age of puberty will be accounted for in the Hereafter.					
8.	Allah S.W.T. is knowledgeable of the movements of the sand particles at the bottom of the ocean.					
9.	Rasulullah's ﷺ teachings are for the advantage and well-being of Muslims only.					
10.	All Islamic laws can be modified to fulfill contemporary needs.					
11.	Rainfall is controlled by angels that have been commanded by Allah S.W.T.					

12.	In emergency situations, Islam allows Muslims to abandon obligatory prayer (*solat*).					
13.	To fully develop their nations, Muslims cannot completely follow Islamic teachings.					
14.	If Allah S.W.T. wills to destroy a place, both Muslims and non-Muslims living there may be affected.					
15.	People who impart beneficial knowledge to others will be rewarded for it in this world only.					
16.	Islamic values are applicable only in certain situations, places and times.					
17.	All deeds (*shari'ah*) performed by Rasulullah ﷺ were guided by revelation.					
18.	Rasulullah ﷺ created laws that were not given to him by Allah S.W.T.					
19.	All human activities must be done for the sake of Allah S.W.T.					
20.	Allah S.W.T.'s rules fulfill all of His creatures' needs.					
21.	Islamic teachings do not fulfill the needs of human beings' natural state (*fitrah*).					
22.	Allah S.W.T. will not test a person who internalizes and practices religion.					
23.	Certain rules ordained by Allah S.W.T. can be violated to achieve success in worldly life.					
