# Supplementary material for: Impact of Perceived Severity of COVID-19 (SARS-COV-2) on Mental Health of University Students of Pakistan: The Mediating Role of Muslim Religiosity
Source: Front Psychiatry. 2021 Aug 2;12:560059. doi: 10.3389/fpsyt.2021.560059 (PMC8365036; doi:10.3389/fpsyt.2021.560059)
Supplement: Supplementary file 3 [file Data_Sheet_3.PDF]

PHQ-4

**Over the <u>last 2 weeks</u>, how often have you been bothered by the following problems?** *(Use "✔" to indicate your answer)*	Not at all	Several days	More than half the days	Nearly every day
1. Feeling nervous, anxious or on edge	0	1	2	3
2. Not being able to stop or control worrying	0	1	2	3
3. Little interest or pleasure in doing things	0	1	2	3
4. Feeling down, depressed, or hopeless	0	1	2	3

<u>Scoring</u>

PHQ-4 total score ranges from 0 to 12, with categories of psychological distress being:

- None 0-2
- Mild 3-5
- Moderate 6-8
- Severe 9-12

Anxiety subscale = sum of items 1 and 2 (score range, 0 to 6)

Depression subscale = sum of items 3 and 4 (score range, 0 to 6)

On each subscale, a score of 3 or greater is considered positive for screening purposes

---

The PHQ scales were developed by Drs. Robert L. Spitzer, Janet B.W. Williams, and Kurt Kroenke and colleagues. The PHQ scales are free to use. For research information, contact Dr. Kroenke at kkroenke@regenstrief.org

Kroenke K, Spitzer RL, Williams JBW, Löwe B. An ultra-brief screening scale for anxiety and depression: the PHQ-4 Psychosomatics 2009;50:613-621.
